# Reconceptualizing the Family to Improve Inclusion in Childhood Disability Research and Practice

**DOI:** 10.3389/fresc.2021.710580

**Published:** 2021-09-10

**Authors:** Michelle Phoenix, Meaghan Reitzel, Rachel Martens, Jeanine Lebsack

**Affiliations:** ^1^CanChild, McMaster University, Hamilton, ON, Canada; ^2^Faculty of Health Sciences, School of Rehabilitation Science, McMaster University, Hamilton, ON, Canada; ^3^Bloorview Research Institute, Holland Bloorview Kids Rehabilitation Hospital, Toronto, ON, Canada; ^4^IWK Strongest Families Research Institute, Halifax, NS, Canada

**Keywords:** family, childhood disability, rehabilitation, family-centered services, inclusion, family systems theory

## Abstract

The World Health Organization's International Classification of Functioning, Disability and Health recognizes that environmental factors impact well-being and life participation for children with disabilities. A primary environment in which children grow and learn is the family. The importance of family has long been recognized in family-centered practice and family-centered research. Although family-centered services and research have been critically explored, the concept of family has received less critical attention in rehabilitation literature. The family construct is due for an updated conceptualization with careful consideration of the implications for childhood disability rehabilitation practice and research. Interrogating the family construct asks questions such as: who is included as a part of the family? Which family structures are prioritized and valued? What is the potential harm when some families are ignored or underrepresented in childhood disability practice and research? What implications could a modern rethinking of the concept of family have on the future of childhood rehabilitation practice and research? This perspective article raises these critical questions from the authors' perspectives as parents of children with disabilities, child focused rehabilitation professionals, and researchers that focus on service delivery in children's rehabilitation and family engagement in research. A critical reflection is presented, focused on how the construct of family affects children's rehabilitation practice and research, integrating concepts of equity, inclusion and human rights. Practical suggestions for children's rehabilitation service providers and researchers are provided to aid in inclusive practices, critical reflection, and advocacy.

## Introduction

As parents (JL, RM), practitioners (MP, MR), and researchers (MP, MR) we acknowledge that children grow and develop in the context of their family. Family is the most immediate and powerful influence on a child's formative years that affects their lifelong trajectory (1). As such we focus on the family as a part of care and generally recognize family-centered service as a preferred framework to guide service delivery for children with disabilities (2). Family-centered services have been described as a philosophy and framework that recognizes families as the constant in a child's life, values parents' knowledge of their child, and their partnership in services (3). Despite the widespread adoption of family-centered service there are reported challenges with implementation, such as difficulties collaborating between parents and service providers (4) and a lack of services that focus on the well-being of the whole family (5). The language used to refer to family caregivers is complex reflecting the informal and typically unpaid role that many family members assume when caring for someone with a disability (6). The concept of family-centered services has been critically examined, recognizing the tension between the family's therapy preferences, cultural norms and expectations, and competing organizational considerations such as service delivery models and funding (7).

Similar to family-centered services, family-centered research has been proposed as a form of patient-oriented research that prioritizes families' interests and perspectives (8). In family-centered research an equal partnership can be created that invites families to engage throughout the research process and share their ideas and critiques in informing the study design, conduct, and knowledge sharing (8). The concept of patient-oriented research has drawn critical attention, raising questions of how to engage patients authentically (9), how to compensate patients (10), how to engage families that are underrepresented in research (11) and regarding the methods that may be used to promote reflexivity throughout the collaborative research processes (12). The conceptual theory underlying patient engagement in health settings and what it means to include ‘the patient voice' has been critically explored (13). Raising these questions has helped to advance practices in this area, generating recommendations that guide researchers to examine and inform their practices to improve quality and inclusivity in research.

While family-centered service and family-centered research have been critically examined, the underlying concept of *family* has yet to undergo formal critical scrutiny as applied in childhood disability research and practice. Traditionally childhood disability scholars and practitioners focused on the child and their health condition in isolation (14). However, application of a biopsychosocial model in the World Health Organization's International Classification of Functioning, Disability and Health (ICF) shifted thinking to a contextual view of the child, recognizing that children's function is impacted by both children's health conditions and their social environments (e.g., families, communities) (15). Sociologists and psychologists have a history of studying the family unit in which family systems theory, social ecological models, and structural-functional theories were used to examine the interdependence of family member identities, roles, and functions (14). These micro and macro-level theories account for how family members construct their individual and family identities as situated within their culture.

Traditionally, the nuclear family structure that represents white heterosexual norms and values was viewed as the typical or even ideal family, including a married mother and father residing with their unmarried biological children (14). Overtime, cultural and legal norms have expanded to recognize other family structures that include adoptive families, divorced families, and step-parent relationships (16). In some cultures, multigenerational families are recognized, with grandparents holding prominent roles in the child's care and home responsibilities (14). There continues to be controversy over whether lesbian, gay, bisexual, transgender (LGBT) couples are recognized by law and allowed to marry, and these families may face additional prejudice and judgement in their communities (17). Little is known about the experiences of LGBT parents who are raising children with disabilities (11, 14). Clinical practice and research with foster-families are complicated by informal kinship arrangements, formalized kinship, or non-kinship foster family status (18). There are family structures that are rarely included in research, such as polyamorous and polygamous families that may face negative judgement and marginalization when seeking health care for their families (17, 19).

It has been said that “it takes a village” to raise a child and this may be especially true for children with disabilities and their families; however “the village” is typically unrecognized in rehabilitation services and research (20). A family's culture and ethnicity may shape “their village” to include cousins, aunts, uncles, grandparents and non-relative members, such as Godparents and these roles may reflect culturally formed expectations regarding financial support, caregiving, and provision of advice (14, 17, 21). People with disabilities (22) and parents of children with disabilities (21) have also included friends and peer support networks in “their village.” These individuals contribute to their well-being, due to an empathetic understanding of peoples' needs, and the availability and willingness to provide physical, emotional, spiritual care, or guidance (21, 22). Despite the high value placed on these relationships and their potentially transactional nature, friendships are not legally recognized with the same rights as family relationships (22).

Family Systems Theory is applied in this article to conceptualize family as:

- a system of individuals that are bonded together through their co-constructed identity as family members,
- people with roles and functions that tie members to one another and influence individual and collective family outcomes,
- the sharing of a social location in a broader environment that (a) shapes families' identity and (b) allows families to shape the culture in which they are embedded.

This article provides a critical reflection on how the conceptualization of family affects rehabilitation practice and research for children with disabilities and their families. The parents on our authorship team (RM, JL) initiated conversation with the researchers (MP, MR) to raise concerns about how family is defined in the childhood disability research and care contexts. They described the high demands placed on mothers, devaluing of non-related family members in their social support networks, and the need to consider research and policy implications (e.g., who is counted on research demographic forms and who qualifies for respite care). We advanced these ideas and generated recommendations through iterative discussions and draft revisions that integrated theoretical concepts and literature with examples from parents', practitioners', and researchers' lived experience.

## How Does the Conceptualization of Family Affect Children's Rehabilitation Practice?

The definition of family used in a clinical setting has major implications for service delivery. At the outset of service, legal guardians, or parents need to consent to a referral, assessment or therapy plan (23, 24). They often provide insight on goals for the child and take responsibility for the implementation of home programs. Parents typically have the right to access information about the child's therapy and progress, for example through the receipt of written or verbal reports from service providers or electronic health records (24). One family member is often called upon to share their child's therapy information with other people who are involved in the child's life, for example their partner, daycare providers, grandparents, or other professionals (25). Often tasks such as providing consent, setting goals, sharing information, and implementing home programs are taken up by the parent who attends therapy, even in dual parent families (25, 26). The heightened demands placed on a parent in single parent families or families that co-parent when only one parent attends therapy have been reported and should be considered when developing a service plan with parents (26).

### How Can We Create Clinical Environments That Are Inclusive of Diverse Family Structures?

1. At the point of intake ask open ended questions to determine how the family members view their family. For example, “Would you mind telling me about your family?” This may provide insightful information about the adults in the child's life, siblings, step-family members, and living situation. Use these insights when completing contact information forms and case history questions.
2. Consider sharing your pronouns to signify that clients and family members are invited to do the same. Consistently use the pronouns that people tell you they identify with when you interact with family members and in clinical reporting. Use gender inclusive language when referring to family members, for example, “does your partner also work during the day, what is their phone number?”
3. Ask families about who they would like to be a part of their therapy and how they would like to communicate with you. For example, a grandparent may work with you because the child is cared for by the grandparents during the day. Can that be accommodated? Would parents like for you to send them progress updates directly or via the grandparents?
4. Use available literature and conversation with clients to reflect on your own biases and heighten your understanding of the care experiences for people whose family structures do not match dominant cultural ideas of family. For example, would individual therapy be more comfortable for a transgender parent than a group program where they may fear and experience judgement from other families?

### What Are the Potential Risks When a Nuclear Family Structure Is Reinforced in Existing Rehabilitation Practices?

If children's rehabilitation service providers do not think critically about how diverse family construction affects their practice, we risk reinforcing existing stereotypes and barriers to service use. Families may feel unwelcome and avoid or delay service use. This may lead to missed opportunities to provide early intervention for children with a cascade of negative outcomes (e.g., missed diagnosis, delayed therapy). In assessment, a holistic understanding of the child's skills, needs, and goals may be lacking if only one parent provides information and other people who are close to the child are not invited to participate. In therapy, service providers who do not discuss family member roles may make erroneous assumptions about the resources and supports that are available to facilitate therapy participation (e.g., bringing children to appointments or doing home practice). Often the burden for sharing information about therapy progress and plans is carried by the adult who attends sessions. A fulsome understanding of who is regarded as family and obtaining necessary consents may allow the service provider to directly share relevant information with each individual. This would reduce the responsibilities for the adult who attends appointments and potentially avoid conflict when sensitive information or recommendations need to be communicated (e.g., a diagnosis).

## How Does the Conceptualization of Family Affect Children's Rehabilitation Research?

The conceptual definition of “family” affects who is recruited, the data collected, and the findings produced in family focused research (16). This is particularly true for families that do not fit traditionally recognized structures, such as, families created through surrogacy or adoption, divorced or blended families, LGBT families, polygamous families, or multigenerational families (16, 17). Researchers who focus solely on the nuclear family may miss opportunities to understand and appreciate broader conceptualization of families that also includes social support networks (e.g., religious communities) and kin who may be relatives or non-relatives (14, 16, 21).

When designing a study protocol, developing inclusion-exclusion criteria, and creating recruitment materials it is necessary to carefully consider the desired sample and to justify the accompanying methodological choices (e.g., sampling strategy, recruitment terminology, venues, and processes). These decisions have tangible implications for the research completed and the potential application of findings. For example, stress and coping in families of children with disabilities are frequently studied, however close examination of this literature demonstrates that it is typically mothers' stress and coping that are documented, with few studies on siblings, grandparents, or fathers (20, 27). When family researchers attempt to include diverse members, such as stepparents, they may face barriers due the stigma associated with particular labels or a presumption that general labels (e.g., sibling) do not include step-siblings or half-siblings (16). In polygamous families, individuals may use invented language, such as “tribal aunt” to signify belonging, and these terms may be unknown and underutilized by researchers (17). By intentionally or unintentionally excluding the people who consider themselves to be family members of a child with a disability, we are missing opportunities to generate data that would inform our understanding of their perspectives and to inform clinical practices.

Research rarely examines the experience of children with disabilities who are raised by parents who are LGBT (11, 17) and siblings and grandparents are often overlooked in the literature. Siblings can be highly involved in therapy and may assume care responsibilities as adults, however research focuses more on parents therapy involvement (28). Research about grandparents of children with disabilities has increased over the past decade, revealing heterogeneity in grandparents' acceptance of the disability, frequent worries for family, high levels of support, and family cohesiveness (29). A paucity of research may reinforce exclusion, under representation and stigma for diverse families, for example families who have undergone divorce or remarriage (16) or polygamous families (17).

Data analysis provides researchers with an opportunity to critique their assumptions, for example are you comparing the family experiences and outcomes to a presumed ideal or normative family type? Are you applying a deficit-based lens to problematize unfamiliar family experiences and could this be reframed from a strengths-based position? For example, research on polygamous families indicates that these families may have challenges and fears about disclosing their relationships to their children and about child custody. However, research also notes the benefits of collaborative parenting in polygamous communities such as, shared resources and increased adults to spend time with the children (17). Engaging members of the community when creating your research question and during data analysis may help to ensure that these strengths-based questions and interpretations are not overlooked (11).

### How Can We Create Research That Is Inclusive of Diverse Family Structures?

1. When designing a study, carefully justify your participant selection criteria and choose language that matches the language used in your target communities. This may require collaboration or pilot testing of your recruitment materials with members of the chosen community.
2. If your research is about families of children with disabilities consider whether your question is inclusive to all family members (e.g., siblings, grandparents) and family structures (e.g., kinship communities, co-parents who do not live together) and justify your decisions. Check whether your data collection forms (e.g., demographic questions) and survey or interview questions allow all family members to contribute data and be included (e.g., how are gender questions worded)?
3. Embed critical reflexivity into your research to interrogate your own position and beliefs and the potential impact on your research. When it is appropriate invite collaboration and critical questioning from people who have a family experience that is different from your own.

### What Are the Potential Risks When a Nuclear Family Structure Guides the Research?

If researchers do not embrace a holistic definition of family that is inclusive of the people recognized as family in the lives of children with disabilities, there is a risk of excluding people from research and reinforcing a narrow understanding of family life. This limited evidence-base will make it challenging to draw from the study findings for use in clinical practice with individuals beyond the client and their mother and father. There will be missed opportunities to understand and reinforce the value and strength in diverse families.

## Discussion

The discussion and recommendations presented thus far were intended to support children's rehabilitation service providers and researchers to (i) develop inclusive practices and (ii) consider the potential risks of maintaining focus on the nuclear family. While we hope that these strategies may be taken up to improve research and practice at the individual level, we recognize that collective advocacy is needed to promote widespread acceptance of diverse families of children with disabilities.

The WHO-ICF highlights three environmental factors that are relevant to this discussion of family: support and relationships, attitudes, and services, systems, and policies (30). Under supports and relationships there is clear evidence to promote the inclusion of families in service delivery and research; however, advocacy may be needed to expand consideration of “who counts” as a family member. For example, do regulatory bodies and privacy guidelines allow grandparents who hold informal guardianship roles to consent to therapy on behalf of a child who resides with them, even if parents hold legal custody? Our clinical experiences as Speech-Language Pathologists and Occupational Therapists in Ontario, Canada suggest that parental consent is required for all treatment decisions, unless legal guardianship has been transferred. Perhaps advocacy is needed to allow flexibility in these circumstances, such that parents could provide a blanket consent allowing grandparents to make therapy related decisions. In research, manuscript reporting guidelines may request that authors justify the congruency between their research question and sample. For example, if your question is about well-being in *parents* of children with disabilities were both mothers and fathers recruited? If it was about *family* well-being, were siblings, grandparents or other family members included? Grant priority funding may be allocated for groups that are often excluded in childhood disability family research, e.g., informal kinship or friendship networks, LGBT parents, polyamorous and polygamous families.

Attitudinal environmental barriers to functioning indicate that children with disabilities and their families are likely to experience disability related stigma and this experience may be heightened for families who hold other identities that are devalued in society (e.g., LGBT parents, families with low socioeconomic status, and racial or ethnic minority families) (14). Implicit bias training for children's rehabilitation service providers and researchers may help people to increase awareness of their own biases about families and to mitigate the potential for negative consequences in client care (31) and research conduct (e.g., how questions are framed and data is interpreted).

Service providers and researchers have a critical role to play in advocating for health services, systems, and policies that promote the inclusion, functioning and well-being of children with disabilities and their families (32). At the broadest level, we should align with Article 5 of the United Nations Convention on the Rights of the Child (CRC), which recognizes that the following people have a duty help children to exercise their own rights over time “…parents or, where applicable, the members of extended family or community as provided for by local custom, legal guardians or other persons legally responsible…” (33) and Article 2, which protects children from discrimination, including that which stems from their parent's or guardian's sex, ethnic or social origin, political or other opinion (33). The WHO-ICF personal factors may aid researchers and service providers in identifying aspects of the individual's background (e.g., age, race, gender) that may interact with a health condition and environment to impact function and participation in everyday life (34). These applications of the CRC and ICF may support service providers' and researchers' efforts to critically examine the identity of clients and families and equitably support their inclusion in services and research.

We recommend that future research be conducted with families, clinicians and researchers to: (i) understand how they conceptualize family, (ii) identify biases in how families experience inclusion in care and research, (iii) promote critical reflection in practice and research, and (iv) advance inclusive practices with diverse families in clinical care and research. To honor the CRC and enact family-centered care and research the ICF personal and environmental factors can be usefully applied to critically examine our conceptualization of children and their families and advocate for full inclusion in rehabilitation services, research, and society.

## Data Availability Statement

The original contributions presented in the study reflect the personal experiences and reflections of the authors, further inquiries can be directed to the corresponding author.

## Author Contributions

MP was primarily responsible for writing this article. The ideas presented were developed by all authors from their combination of lived experience as parents of children with disabilities, clinical experience in children's rehabilitation, and academic research. All authors have had an opportunity to review, revise, and approve the article. Authors share responsibility for the information presented in this article.

## Funding

The authors are grateful for the Rowe Scholarship that supports the PhD work of MR and funding from The Hallman Foundation that supports the research of MP.

## Conflict of Interest

The authors declare that the research was conducted in the absence of any commercial or financial relationships that could be construed as a potential conflict of interest.

## Publisher's Note

All claims expressed in this article are solely those of the authors and do not necessarily represent those of their affiliated organizations, or those of the publisher, the editors and the reviewers. Any product that may be evaluated in this article, or claim that may be made by its manufacturer, is not guaranteed or endorsed by the publisher.
